# The Development of Temporal Concepts: Linguistic Factors and Cognitive Processes

**DOI:** 10.3389/fpsyg.2018.02451

**Published:** 2018-12-05

**Authors:** Meng Zhang, Judith A. Hudson

**Affiliations:** Department of Psychology, Rutgers University, The State University of New Jersey, New Brunswick, NJ, United States

**Keywords:** temporal concepts, temporal language, conceptual development, language development, temporal perspective

## Abstract

Temporal concepts are fundamental constructs of human cognition, but the trajectory of how these concepts emerge and develop is not clear. Evidence of children’s temporal concept development comes from cognitive developmental and psycholinguistic studies. This paper reviews the linguistic factors (i.e., temporal language production and comprehension) and cognitive processes (i.e., temporal judgment and temporal reasoning) involved in children’s temporal conceptualization. The relationship between children’s ability to express time in language and the ability to reason about time, and the challenges and difficulties raised by the interaction between cognitive and linguistic components are discussed. Finally, we propose ways to reconcile controversies from different research perspectives and present several avenues for future research to better understand the development of temporal concepts.

## Introduction

Time is an essential dimension of the universe. The concepts of past, present, and future are important mental constructs for structuring experiences. We live in the ever changing present, and our experience of past, present, and future keeps shifting (Harner, 1982). Adults have a dynamic and flexible temporal perspective, which allows us to organize experiences and navigate through time mentally, but when do children acquire the concept of time? To grasp the abstract idea of time is not easy. A concept of time depends on the acquisition of many time-related abilities such as understanding and being able to talk about time, being able to distinguish the past, present, and the future, and reasoning about the sequence of events. Researchers studying both cognitive development and language acquisition have investigated children’s understanding of time. However, the findings from these lines of research are not consistent. Because understanding time is a multi-facet competence that draws upon various cognitive and linguistic faculties, reconciling research findings from these different perspectives will help further our understanding of the roles of cognition and language in understanding time. This paper reviews research on children’s understanding of time, focusing on the cognitive and linguistic components involved in early development.^1^ In particular, conflicting results from language development studies and studies addressing cognitive processes are discussed, as well as theoretical issues about the role of language in the development of time concepts. Because children’s cognitive abilities and linguistic capacities are interdependent, practical issues about how to measure each component individually are also considered. Finally, directions for future research to resolve theoretical and practical issues are proposed.

## Conflicting Evidence From Past Research

The limited literature investigating the emergence of temporal concepts comes from two research lines focusing on children’s temporal language acquisition and their temporal cognitive processes, respectively. Psycholinguistic researchers claimed that the separation of event time from speech time indicates children’s emerging concept of time and their usage of tensed verbs is evidence of a grasp of the basic distinctions between past, present, and future by age 3 (Weist, 1989). However, researchers focusing on temporal cognition concluded that 4- and 5-year-olds do not yet understand the distinctions between the past, present, and future properly (Friedman, 2003). What is the evidence for these conclusions and how can they be reconciled?

### Acquisition of Temporal Language

Time is encoded in language in many ways. Language is the primary medium through which notions about past and future events are transmitted (Harner, 1982). In English, many devices, such as aspect, tense, and temporal adverbs, are used to denote time and code time-related characteristics of actions (Klein, 2009). For example, aspect delineates the internal contour of the event itself, whereas tense and temporal adverbs denote the position of an event on a timeline. Developmental psychologists have argued that the emergence of temporal markers in children’s language indicates changes in their understanding of time (Weist, 1989; Busby Grant and Suddendorf, 2011).

In tensed languages, three important points in time are encoded in speech (Reichenbach, 1947). Speech Time (ST) is the time point of the act of speech. Event Time (ET) is the time when the event occurred, and Reference Time (RT) indicates the speaker’s temporal vantage point. It is particularly clear when RT does not coincide with ST and ET, as in the case of the past perfect tense (e.g., in “Peter had gone,” RT is between ET and ST) and the future perfect tense (e.g., in “Peter will have gone,” RT is after both ST and ET). Based on Reichenbach’s theoretical work and observations of language acquisition, Weist (1989) proposed a four-system model of children’s temporal language development, with each system reflecting a different level of competence. The first system is the ST system used by children from 12 to 18 months. Children’s speech at this stage focuses on here-and-now. It does not include tense, aspect, or modality. Between 18 and 24 months, children begin to use past tense to mark an event anterior to speech time and to use future tense to mark an event posterior to speech time. This corresponds to the ET system, where ET is expressed separately from ST. Later, between 30 and 36 months, children start to use temporal adverbs to indicate when an event occurs, which corresponds to the restricted reference time (RT_r_) system. For example, a child might say, “Yesterday I was in Lodz” (Weist, 1989, p. 108). Compared to utterances from ET system, e.g., “I was in Lodz,” utterances from the RT_r_ system contain both event time (i.e., past tense) and reference time (i.e., yesterday) and both are referenced in contrast to speech time. The last system is the free reference time (RT_f_) system, emerging between 36 and 52 months. Compared to the RT_r_ system, children are now capable of manipulating RT, ST, and ET to freely express more temporal configurations. They can use the temporal prepositions “before” and “after,” perfect tenses, and even temporal clauses – for example, “While this one is playing [RT], that one will be playing [ET]” (p. 105). Weist believed that the separation of ET from ST (i.e., the use of tense) indicates an emerging concept of time and that reference to specific non-present time points (i.e., the use of temporal adverbs) indicates a developing temporal framework. The development from RT_r_ system to RT_f_ system relates to more complicated cognitive processes such as temporal decentering and relational reasoning.

In support of Weist’s model, studies focusing on children’s natural language production reveal a haphazard use of inflected verbs from 21 to 22 months (Nelson, 1989). From 22 to 24 months, children develop a present-past-progressive system, which first reflects a contrast between now and not-now, and later takes on direction by specifically coding pastness (Nelson, 1989). However, production of verbs with visible tense marking is rare in 2- to 3-year-olds’ spontaneous speech (Valian, 1991). Even when they are asked to imitate adult’s utterance of past tense, only 2% of verbs are past tensed by 2-year-olds with low mean lengths of utterance (MLU, from 1.5 to 2.5 words) and 14% of verbs are past tensed by 2-year-olds with high MLU (2.5 to 4.6 words) (Valian and Aubry, 2005). Instead of verb inflections, the future time of an action is conveyed in English by a set of modal auxiliaries such as *will, shall, may, must*, and *can*. Research by Ames (1946) and Harner (1981) found that children first spontaneously produced words such as *gonna* and *in a minute* to denote future at age 2; later they used *is gonna/is going to* predominately when referring an action that was just about to happen.

Children’s elicited language production indicates that they are able to use past tense and future verb forms quite accurately by age 3. For example, Harner (1981) demonstrated actions within a short timescale (e.g., a doll went down a slide) to 3- to 7-year-olds and asked them *Tell me about this one* while pointing to either the toy that had completed the action or to an identical toy that always did the same thing; children were asked to either describe what the first toy had done or what the other toy was going to do. Three-year-olds were able to distinguish past and future actions, and the majority of their responses contained past tense (70%) and future verb forms (87%). Other researchers elicited children’s temporal language in describing events over a relatively longer timescale by simply asking questions such as, *What are you going to do tomorrow?* and *What did you do yesterday?* Three-year-olds were able to answer the tomorrow question with the appropriate verb form, *gonna*; 4-year-olds were able to answer the yesterday question using a past tense verb (Ames, 1946; Busby and Suddendorf, 2005).

Language production data indicates that children also begin to use temporal adverbials between 2 and 3 years of age (Ames, 1946; Weist, 1989; Pawlak et al., 2006). Ames (1946) observed 1.5- to 4-year-olds’ spontaneous language production and found that references to the present (*today*) emerged around 24 months, references to the future (*tomorrow)* appeared around 30 months, and references to the past (*yesterday*) appeared around 36 months. Similarly, a longitudinal study (Pawlak et al., 2006) found that children produced *today* and *tomorrow* earlier than *yesterday*. However, although young children are able to produce temporal adverbs in the appropriate sentence position, their actual temporal references may be inaccurate (Bloom, 1970; Busby Grant and Suddendorf, 2011). For example, parents evaluated their 3- to 5-year-olds’ use of temporal terms such as *yesterday* and *tomorrow* as less appropriate than their use of more general terms such as *now, soon*, and *later* (Busby Grant and Suddendorf, 2011).

These findings suggest that although children are able to produce temporal terms at 2 and 3 years, their usage may not always be appropriate. Nelson (1991) proposed that very young children have a basic grasp of time and temporal language, but their understanding is still limited as compared to older children. This interpretation raises the question of what temporal components children understand when they first use temporal language. This question has not been fully addressed, but one approach has been to examine children’s comprehension of temporal language independent of their production of temporal language.

Temporal language comprehension studies have shown that 2- and 3-year-olds understand how tense is used to denote the past and future, but not how more precisely temporal adverbs locate events in time. For example, Herriot (1969) found that 3-year-olds used inflections and modal auxiliaries to correctly identify past and future actions, even with novel verbs. He presented completed and not-yet-begun actions to children using movable toys (one at the starting point of an action, and the other at the ending point). A novel verb (e.g., gling) was used to describe the action and children were asked, *Which one is going to gling?* and *Which one has glinged?* With even younger children, Valian (2006) demonstrated familiar actions such as tying shoes (e.g., one tied and the other about to be tied) and asked children either, *Show me the one I did tie* or, *Show me the one I will tie*. Two-year-olds successfully distinguished the auxiliaries *will* and *did* for future and past actions. Adding temporal adverbials to questions, such as *before* or *already* for the past and *in a second* or *next* for the future improved 3-year-olds’ performance, but not 2-year-olds’ (Wagner, 2001; Valian, 2006).

Understanding how temporal adverbs are used to represent, localize, and organize events in time is more difficult than understanding how verbs denote completed or future events. Weist et al. (1991) compared children’s understanding of sentences referring to past and future ET using only tense (e.g., *The girl threw/will throw the snow ball*) to their understanding of sentences referring to the RTr framework using both tense and temporal adverbs (e.g., *The girl will dance tomorrow/in a while*). They found that children could parse the temporal relation coded in the ET system (using tense) at 2.5 years, much earlier than they could parse temporal relations coded in the RT_r_ system (using tense and adverbs), which was not achieved until 5.5 years.

What makes sentences with both tense and temporal adverbs more difficult? One possibility is that younger children simply do not understand temporal adverbs and find sentences containing temporal adverbs to be confusing. To test this possibility, researchers examined children’s understanding of common temporal adverbs, such as *yesterday, today*, and *tomorrow*. In contrast to results from language production research, results from comprehension studies suggest that children’s grasp of *yesterday* (referring to the past) appears before that of *tomorrow* (referring to the future). Harner (1975) assigned toys to 2- to 4-year-olds to play with on successive days (i.e., yesterday, the testing day, and tomorrow) and asked children to show a toy *from yesterday* and a toy *for tomorrow*. She found that 2-year-olds barely understood either *yesterday* or *tomorrow*, 3-year-olds performed better on *yesterday* questions, and 4-year-olds understood both *yesterday* and *tomorrow*. Zhang and Hudson (2018) explicitly tested children’s understanding of the relational underpinnings of *yesterday* and *tomorrow*. They presented 3- to 5-year-olds pairs of pictures of objects with visible changes of state (e.g., a carved pumpkin and an intact pumpkin) and sentences referring to an action about the target object (e.g., *I carved the pumpkin yesterday* or *I’m gonna carve the pumpkin tomorrow*). Children were asked temporal questions such as *What does it look like now*? Compared to Harner’s task, this task not only requires children’s understanding of *yesterday* and *tomorrow* as distinct categories but also their understanding of the underlying temporal relations between the past and the present and between the future and the present. Similar to other comprehension studies, they found that children answered questions about *yesterday* more accurately than they did for questions about *tomorrow*.

Thus, there seems to be a lag between children’s production of temporal language and their comprehension of relational temporal language. Language production studies showed that children begin to use tense around 2 years old and begin to use temporal adverbs around 3 years old. Language comprehension studies showed that children were able to parse temporal relations from tense around 2 to 3 years, but they could not understand the temporal relations coded by temporal adverbs even at age 4 or 5. Such production-comprehension asymmetries have been observed in many studies of children’s language, with comprehension typically found in advance of production (Clark, 1995). Delays in comprehension compared to production are found in children’s mastery of pronouns, scalar implicatures, aspect, deictic references, and other linguistic forms (Hendriks, 2014). For example, research showed that children produced pronouns (*him, her*) from age 2 or 3, but the adult-like comprehension of pronouns was usually not found before age 6 (Sekerina et al., 2004).

Explanations for discrepancies between language comprehension and production come from four perspectives. First, the grammatical account claims that children’s immature use of syntactic direction-sensitive constraints causes delays in comprehension. Production begins from the input of meaning to the output of optimal form, whereas comprehension begins from the input of form to the output of optimal meaning. Comprehension lags production because young children cannot compute the speaker’s alternative perspective. They have to acquire a Theory of Mind or develop greater processing capacity to be able to compute constraints from both the hearer’s and the speaker’s perspectives (van Hout, 2007). Second, the interface account emphasizes the cognitive resources needed for processing and integrating linguistic knowledge, discourse, and situation information (Hendriks and Koster, 2010; Hendriks, 2014). Working memory and cognitive control are required to keep multiple interpretations in mind during comprehension (Reinhart, 2004). Third, the pragmatic account proposes that children lack pragmatic knowledge (e.g., knowledge of implicature, reference, deixis, discourse structure, etc.); they may not yet be aware of the subtleties involved in using certain words or grammar, which makes comprehension more difficult. Fourth, the delay of comprehension may be due to the testing context (Grimm et al., 2011). Most comprehension tasks take place in non-naturalistic and highly controlled situations and test sentences are often presented with minimal context. Children lack contextual knowledge of the testing situation that would support their comprehension of the presented sentences (Papafragou and Musolino, 2003).

With regard to comprehension and production of temporal language, this asymmetry may occur because the understanding of relations conveyed by temporal language not only requires a basic understanding of the meaning of temporal markers in language, but also the ability to mentally represent temporal relations between events, which may develop later. For example, a basic grasp of temporal markers may only involve a discrimination of them (e.g., *yesterday* refers to different things from that of *tomorrow*) and a sense of their sentential distribution, which seems enough for young children to produce utterances with temporal markers. Bloom (1970) case study showed that a 30-month-old was able to produce sentences with temporal terms such as *today, next Monday*, and *last night* in appropriate positions, but the actual temporal references of these adverbs were inaccurate except for the term *now*. A mature understanding of temporal markers involves a differentiation of the past and the future in terms of sequence, causal relations, distance to the present, and so on. These distinctions require more cognitive processes in order to represent events, to mentally manipulate event representations, and to map representations and relations to linguistic expressions. With the development of these cognitive skills, children’s temporal understanding becomes more refined and their use of temporal language production also becomes more accurate and precise, as Weist described in his RT_f_ system, when children are able to manipulate and express multiple temporal relations.

### Children’s Temporal Representation of Events

Conceptualizing time and mapping language onto temporal constructs involve a number of cognitive processes, including event representation, memory, and reasoning. Children’s implicit and explicit understanding of time derives from their representation of events, including the expected sequence of components within particular events, the representations of sequences of multiple events, and eventually, the localization of events in time (Nelson, 1991). McCormack and Hoerl (1999, 2001) distinguished two frameworks, perspectival and non-perspectival, that can be used for representing the temporal location of events. Temporal representations within a perspectival framework locate entities/events relative to one’s own position/point of view, for example, describing an event as days from present. In contrast, representations within a non-perspectival frameworks locate entities/events independent of one’s position/point of view, for example, describing an event on a given calendar date. Concepts of the past, present, and future are included in the perspectival framework because the past and future are defined from the vantage point of the present. With a stable but ever changing present, our temporal perspective is dynamic and the contents of past, present, and future keep shifting (Harner, 1982). In this review, we focus on children’s acquisition of the perspective framework of time, specifically their acquisition of tense and temporal adverbials as discussed in previous section, and their ability to differentiate the past, the present, and the future as discussed in this section.

Friedman and colleagues (see Friedman, 2003, 2005 for reviews) conducted many studies investigating children’s representation of a temporal framework ordered with distinct categories for past, present, and future events. In most of these studies, children were asked to judge the past–future status and temporal distances of events in verbal and spatial (timeline) tasks. Friedman consistently found that children often confused the past–future status; they judged impending events as being a short time ago and recent past events as belonging to the near future (Friedman et al., 1995; Friedman and Kemp, 1998). For example, most 4-year-olds responded “yes” to the question, “Is Halloween coming soon?” in the weeks after the holiday (Friedman, 2000). Friedman argued that this confusion comes from a distance-based process of temporal differentiation, in which distance to the present is a salient cue for children to locate and differentiate the time of events.

For more familiar daily events (such as waking up in the morning, eating breakfast, lunch, dinner, going to bed), Friedman (2002) found similar past–future confusion. When tested after breakfast and before lunch, about 75% of 4-year-olds judged that lunch would occur in the future, but only 50% of them correctly judged that breakfast had occurred in the past. Similar confusions in 3-year-olds’ temporal judgments were reported by Tillman et al. (2017) in a study about representions of familiar events using a timeline. This limited ability in discriminating past–future status does not only appear for events with cyclic patterns. Friedman (2003) found that 4-year-olds failed in judging the temporal locations of autobiographical events provided by their parents.

Busby Grant and Suddendorf (2009) tested children’s temporal differentiation by using a past timeline and a future timeline separately. Three silhouettes of a person were placed at the appropriate points along the timeline to indicate the passage of time. Children were told that a larger silhouette indicated further in the future (e.g., “this is a picture of a bigger person, like when you are going to be a bit bigger than you are now”); a smaller silhouette indicated longer ago in the past (e.g., “this is a picture of a smaller person, like when you were a bit smaller than you are now”). Children were asked to locate daily, annual, or remote events along the timeline. For example, the experimenter showed children a picture of toothbrush and asked “when did you last clean your teeth? Was it a little time ago, a long time ago, or a really long time ago? Point to where you think it should go.” Three-year-olds discriminated times of past events but failed to discriminate times of future events. Four-year-olds performed well for past events, and differentiated daily events from more remote future events. Five-year-olds differentiated both past and future events across all temporal distances. Hudson and Mayhew (2011) also found that after age 5, children were equally accurate in locating past and future events on a timeline. They showed children pictures of events, either depicting someone else (e.g., “This girl is going to the dentist tomorrow”) or themselves (e.g., “When did you go to Sari’s birthday party?”), and asked them to place the picture on a timeline made of rectangles representing days. Similarly, they found regardless of the effects of temporal distance, a differentiated sense of the past seemed to emerge earlier than a differentiated sense of future.

The findings from this line of research suggest that children’s ability to distinguish between past and future events is not as firm as would be expected from studies of temporal language comprehension and production. One explanation for this discrepancy between findings from language-based and timeline-based studies of children’s temporal understanding is that in temporal judgment studies using spatial representations of time, the distance of events to the present is very salient. Young children may focus on the distance of an event to the current time point without considering whether events have already happened or have yet to happen. Another issue with the timeline methodology is that the direction of past and future and the scale of distance vary considerably across spatial tasks. For example, Friedman and colleagues (see Friedman, 2003, 2005 for reviews) used tasks in which time was represented as a road stretching ahead in front of the viewer; whereas other researchers (e.g., Busby Grant and Suddendorf, 2009; Hudson and Mayhew, 2011; Tillman et al., 2017) used horizontal time lines where time flowed from left to right. The variations in spatial representation of temporal direction and the saliency of temporal proximity to the present that are entailed by timeline-based measures are not an issue in language-based measures. This may contribute to the discrepant results from these two methods.

Judging and locating events on a timeline measures children’s sequential representation of events which is the cognitive foundation for other types of temporal reasoning, such as sorting out the relations between events in the past, present, and the future. Research has shown that children understand basic sequential relations by age 3. For example, Carni and French (1984) told children stories about familiar events with pictures of events in the story and asked them what happened *before* or *after* a specific action. They found that 3-year-olds reliably distinguished between sequential relations of before and after given this highly supportive context.^2^ Similarly, Fivush and Mandler (1985) presented children pictures of familiar events such as going to the supermarket, and unfamiliar events such as going to parachute jumping. After a careful view of all the pictures, children were asked to put randomly ordered pictures in sequence. They found that 4-year-olds were able to reconstruct the temporal sequences of many familiar events. In general, forward temporal reasoning is easier than backward temporal reasoning for children (Tillman et al., 2015; Zhang and Hudson, 2018). Familiar events in forward order are the easiest to sequence, followed by unfamiliar events in forward order, familiar events in backward order, and finally, unfamiliar events in backward order (Fivush and Mandler, 1985).

Moreover, the temporal organization of an event is also a function of how well the mental representation of the event is encoded (Mandler, 1986). For events with a clear goal, outcome, and internal relationships, event representations are easier to be established and the temporal sequences of event components are encoded automatically during initial construction. Causation is one internal relationship that connects events or event components. Physical causes precede effects; therefore causation inherently contains temporal sequence. Using an elicited imitation paradigm, Bauer and Shore (1987) and Bauer and Mandler (1989) showed that children as young as two recalled events with causal relations better than those lacking causal relations, and when causal relations were interrupted, children were still able to organize their recall around causal relations.^3^

A sense of the past and future not only involves judging events as belonging to the past or the future, but also an understanding of the conceptual relations between the past and future. For example, a past event, but not a future event, could physically affect the present state of affairs. The past, but not the future, can be known; the future, but not the past, can be altered. Although children’s ability to reason about temporal and causal relations develops with age, 3-year-olds already understand that physical causes precede their effects (Gelman et al., 1980). The inherent sequence within causation contributes to children’s understanding of conceptual relations between the past and present. Povinelli et al. (1999) presented children with videos and verbal descriptions of two past events in which they just participated such as hiding a puppet. Children as young as 4 years were able to find the puppet in its current location, indicating that they understood that the very recent past events causally determined the present. With age, children’s grasp of causal relations between past, present, and future becomes flexible and applicable in different contexts. Busby and Suddendorf (2010) investigated children’s temporal reasoning by describing two short vignettes to children: one about a character who acquired an object (e.g., a balloon) or knowledge (e.g., a name) in the past, and the other about another character acquiring that object or knowledge in the future. Children were asked which character currently possessed the object or knew the fact. They found that 5-year-olds were able to distinguish past and future changes in both physical and mental states. Friedman (unpublished, cited in Friedman, 2003) also reported that 6-year-olds could articulate the causal relation between both the past and the present, and between the future and the present. This conceptual understanding of the past and future in 6-year-olds correlated with their judgment of the past–future status for autobiographical events, supporting the idea that causal understanding underlies children’s temporal reasoning.

A crucial ingredient of temporal reasoning is the ability to envisage events from multiple temporal points of view, referred as temporal perspective taking (McCormack and Hoerl, 2001). It allows individual to switch back and forth from different vintage points of time, i.e., temporal decentering. In temporal reasoning tasks, children are often presented with events that happened at a given time point and are asked to reason about situations based this information. Temporal decentering is involved because the question and the given information are about different time points. Children must retain the relevant information in memory and mentally travel from Time A that was specified in the provided information and infer its effect or implication for Time B. For example, to determine whether a character, who is about to get a balloon tomorrow, has a balloon now, children need to first decenter from the present and project themselves to tomorrow, when the character is acquiring the balloon, take the perspective of this time point, and recall that the question asked about events that happened before this point, then switch back to the present, and respond to the question. Temporal perspective switching and temporal decentering are the keys to this temporal reasoning process.

Moreover, because temporal reasoning is based on a concept of time as a successive series of causally interdependent states, it plays an important role in many higher order cognitive processes, such as planning and problem solving. McColgan and McCormack (2008) examined 3- to 5-year-olds’ temporal-causal reasoning in searching and planning. In their search task, children observed a puppet walking through a miniature zoo, passing different cages and taking a Polaroid picture at the kangaroo’s cage. At the end of the visit, the puppet noticed the camera was missing. While viewing the photo of the kangaroo, children were asked where in the zoo the camera might have been lost. In their planning task, the same scenario was used, and children were told that a puppet wanted to visit the zoo and take a picture of the kangaroo. Children were asked to pre-position the camera in the zoo so that the puppet could take the desired picture when passing by the kangaroo’s cage. To make an appropriate choice, children had to combine knowledge about the temporal order of events with causal evidence (in the search task) or knowledge (in the planning task, the camera is a prerequisite for taking pictures). Four- and 5-year-olds, but not 3-year-olds, succeeded in the search task. Only 5-year-olds performed well on the planning task, whereas 3- and 4-year-olds’ performance was at chance. Using a closely matched control task requiring mere updating, Lohse et al. (2015) found younger children succeeded in the control task but not the search task. These findings indicate that temporal-causal reasoning is qualitatively different from simple updating. It seems to emerge at around 4 years of age and continues to develop in children from 5 to 6 years old.

In summary, studies focusing on temporal language indicate that children are able to distinguish past and future at 2–3 years, but studies focusing on temporal cognition show that children at age 4 and 5 years still display past–future confusion; they are not capable of reasoning about the past and future until age 5. This controversy may relate to the different methodologies employed in each line of research, for example, production of tense was taken as an indicator of temporal concepts in psycholinguistic studies whereas differentiation of past and future events and their effects was considered as temporal understanding in cognitive developmental studies. However, more importantly, the controversy draws attention to the mental processes involved in mastering temporal language and making temporal judgments and reasoning, and raises crucial questions such as: Does children’s early use of temporal language indicate temporal understanding? How much do temporal judgment and temporal reasoning tasks tell us about children’s temporal concepts? With both being closelsy involved in conceptual development, how can we identify the mental processes for temporal language and those for temporal cognition? Furthermore, how can we tease apart linguistic and cognitive processes in temporal reasoning tasks? By addressing these questions, we can begin to disentangle the linguistic and cognitive components in the conceptualization of time. Theoretical issues concerning the role of language in the development of temporal understanding and practical issues concerning how to assess cognitive and linguistic components separately are discussed in turn below.

## Is Language Necessary for the Very Formation of Temporal Concepts?

Our concepts of time are abstract; they are primarily communicated via language. The relationship between language and concept formation or cognition in general has been discussed by many theorists, including Chomsky, Piaget, Whorf, and Vygotsky. Piaget and Vygotsky focused on the effect of language development on changes in thought. They both assumed that thought and language are distinct representational systems. Piaget (1968) held a cognitive determinism view. He claimed that children’s grasp of word meanings changes with development and reflects underlying changes in thought. Language is necessary but not sufficient for the construction of logical operations. Both language and logical operations depend on non-linguistic intelligence. The intellectual unfolding of children’s mind sets the pace for their language development. Vygotsky (1962) emphasized the interaction between language and thought. He proposed that language augments children’s prelinguistic cognitive abilities; it gives children the control over their own mental processes such as directing attention, selecting a course of thought, and formulating mental plans. Vygotsky also emphasized the impact of social interaction and cultural symbol systems on language and cognitive development. Taking a Vygotskian perspective, Nelson (1991) argued for mutual influences between language, world knowledge, and the sociocultural context. She considered language and cognition as interactive systems with cognitive development inseparable from language. The interdependency between cognition and language is especially salient in children’s acquisition of temporal concepts.

What role, then, does language play in constructing temporal concepts? Is language necessary for the very formation of these concepts and not merely for their expression? Do pre-linguistic children have some basic temporal understanding? Although researchers (O’Connell and Gerard, 1985; Bauer and Mandler, 1992) have found evidence of sequential understanding in 11-month-old infants using an elicited imitation paradigm, non-linguistic concepts of past and future are very difficult to assess. Nelson (1989) proposed four logical possibilities with respect to the relation between the linguistic expression of time and the mastery of time concepts: (1) Concepts of past, present, and future are innate and will be expressed in language when language development has reached a particular level; (2) Concepts of past, present, and future are an inherent part of the human conceptual system, but this system matures independent of linguistic development; (3) Concepts of past, present, and future are constructed. Temporal language may facilitate the construction of the temporal systems by flagging potential distinctions, but the concepts are not wholly dependent upon linguistic expression; (4) Concepts of past, present, and future are dependent upon language expression for their construction.

Nelson (1989) longitudinal study of a 2-year-old child’s (named Emily) pre-sleep monologs provides data to support the view that temporal concepts are constructed in response to linguistic coding (possibility 3). Linguistic coding of temporal concepts and relations emerged relatively late in Emily’s speech, but correlated with the development of many related notions such as far and near, past, future, general event knowledge, frequency, contingence, and possibility. Further, many temporal adverbs, prepositions, and conjunctions appeared simultaneously in Emily’s speech, which, according to Nelson, helped build a system of mutually defining temporal and causal relations and guide the acquisition of temporal concepts. These findings suggest that temporal language facilitates the construction of the temporal systems. Moreover, compared to relative concepts of time, such as temporal perspectives (past, present, future), temporal sequence, duration, and speed of events, arbitrary concepts tied to conventional time systems, such as seasons, months, days of a week, hours, require direct teaching by the language community (Nelson, 1991). In other words, children need explicit discussion and teaching from adults to acquire meanings of such lexical terms. For example, Tillman and Barner (2015) found that preschoolers had little to no knowledge of the absolute durations encoded by duration words (e.g., second, minute, hour, day, etc.). This knowledge is learned when they acquired the formal definitions for the words.

However, many commonly used temporal terms, such as *morning, afternoon, night, yesterday, tomorrow*, etc., are not directly taught to children. How do children learn these? Everyday communication between parents and children often contains a variety of temporal terms, for example, *Tomorrow we’re going on a trip, Remember last week we were at grandma’s house*, etc. These temporal terms (e.g., *tomorrow, last week*, etc.) refer to pseudo-objects whose meanings are not clear to children initially. They may serve initially as placeholders, which contain little meaning content, but have strong associations with specific contexts. These contexts are situations in which the terms have been used by parents. Children hold basic representations for the placeholders, for example, a rough idea about the domain referred by temporal terms and the distribution of temporal terms in a sentence. At this point, children acquire the forms of words from the discourse context but with little conceptual underpinnings. Their early use of temporal terms is limited to the associated contexts and oftentimes inaccurate. For example, they may produce sentences with temporal adverbs (e.g., *yesterday, tomorrow*, etc.) in appropriate sentence positions but refer inaccurately to time points (Bloom, 1970). In other words, the reference of their temporal linguistic expressions does not match the actual event time that they intend to express. Nelson (1991) called this “use before meaning;” it is consistent with Vygotsky’s account of language acquisition in which “grammar precedes logic” (Vygotsky, 1962, p. 127). Thus, early use of temporal words is not necessarily evidence of early temporal understanding.

Parents’ feedback and children’s own experience of events allow them to update and refine the meanings of the linguistic forms. As contexts entailing temporal language accumulate and diversify, children’s grasp of temporal terms gradually becomes decontextualized. They can now generalize the terms to novel situations. During this process, temporal language facilitates the construction of the conceptual temporal systems by introducing new ideas and flagging potential distinctions, such as using the term *yesterday* to refer to any not-now event. At the same time, children’s level of cognitive ability also affects how much children benefit from hearing and using temporal language (Sachs, 1983). For example, Nelson (1977) observed a 3-year-old who mistakenly reversed the order of past events by describing the recent event first and the second recent event next, so on. This narrative pattern indicates the cognitive difficulty of decentering oneself to a non-present point and following the temporal sequence from there. Children’s cognitive readiness for flexibly switching temporal perspectives, and for coordinating and manipulating mental representations of events, affects their use and understanding of temporal language in narrative discourse.

How much conceptual understanding of time can be inferred from children’s natural language production? Nelson (1989) argued that appropriate production of temporal terms might not indicate a genuine understanding. Children may use the terms meaningfully in a subset of contexts where adults use them, or simply copy adults’ usage in a particular context. For example, in Emily’s pre-bed monolog, she used the expression “just a minute” to request her father to rock her in the crib (“Daddy came in just a minute and rocked me,” Nelson, 1991, p. 303). This expression was only used in this context at that time and it was the same context that her father used (he usually responded to Emily’s request to be rocked with “I will rock you for just a minute,” Nelson, 1991, p. 303). Because of the strong association between the use of “just a minute” and the crib-rocking context, Emily’s production of the phrase was not underpinned by a genuine comprehension of meanings (either a duration of 60 s or “a little while” in general). Contexts that entail children’s active involvement or interest (e.g., Emily desired to be rocked in crib), as well as repetitive interactions associating the context with a small set of temporal terms, seem to incubate the production of those terms. Such production is context dependent; it is an important mid-point in the continuum of concept mastery from “not at all” to “full command”.

For these reasons, researchers should be cautious in making conceptual inferences from language production data. For example, whether children’s initial use of past tense encodes ordered time relations or aspectual features is under debate. The aspect-before-tense hypothesis claimed that children initially used past tense to mark the completedness of an action, not the time of the action (Bronckart and Sinclair, 1973; Antinucci and Miller, 1976). Therefore, children could not be said to understand the notion of pastness until they used past tense for both continuous, non-goal-oriented actions and completed, goal-oriented actions. However, other researchers provided evidence suggesting that English-speaking children were able to use past tense to refer a variety of past events, not just to goal-oriented ones with completive aspects (Kuczaj, 1977; Di Paolo and Smith, 1978; Sachs, 1979). They also argued that despite children’s earliest tendency to use past forms in their own speech to signal a “present completedness of a past action,” they might understand references to past events in the speech of others (Harner, 1982, p. 153). Children’s production and comprehension of tense should be analyzed in conjunction with consideration of action types (goal- vs. non-goal-oriented) in making inferences about their understanding of the concept of past.

Research directly addressing the role of language in forming concepts of time is very limited, but the influence of language has been addressed for many other aspects of conceptual development. For example, count nouns are considered “invitations” to children to form categories (Waxman and Markow, 1995). They serve as labels for concrete objects (or sets of concrete objects) and help children form theoretical kinds in mind (Gelman and Coley, 1991). Researchers (Waxman, 1991, 2004) believe that language facilitates children in establishing conceptual organizations such as categorical hierarchies. For young children, nouns highlight higher-order category relations (e.g., animal, plant) and adjectival phrases mark specific, lower-order distinctions (e.g., edible mushrooms, poisonous mushrooms). A majority of the word-learning literature focuses on the mapping process between a conceptual category and its linguistic label. Several conceptual bases or initial constraints, such as the whole-object, taxonomic, and mutual exclusivity assumptions, have been shown to be useful in solving the inverse problem of mapping (Markman, 1991). Beyond categorization, language is an important instrument for children to acquire relational concepts. The use of common labels for relational roles (e.g., *daddy, mommy, baby*), the possession of relational verb (e.g., *buy* and *sell, come* and *go*), relational adjectives (e.g., *high* and *low, more* and *less*), and even names for relations (e.g., *same* and *different*) provide representational tools, which make the restricted implicit understanding of relations into a more powerful explicit one (Gentner, 2003; Christie and Gentner, 2014).

Similarly, children begin to produce *no* and *not* between 15 and 27 months, but their grasp of the full range of meanings as a logical operator that flips the truth-value of a proposition comes later (Feiman et al., 2017). This lag echoes the one between production and comprehension of temporal terms, and is also evident in children’s acquisition of mental state words. Researchers (Nelson, 1996a; de Villiers and de Villiers, 2003, 2014) investigating the connection between mental state words and the development of Theory of Mind (ToM) noticed that children started to use language about mental states, such as verbs of desire, belief, and knowledge at age 3, around the same time they showed their ability in monitoring others’ mental states (Bartsch and Wellman, 1995). Although children’s use of mental state terms may not be interpreted as having the same meanings that adults attached to them, having labels for abstract mental states and being able to talk about minds make their representations of mental states more portable.

We can draw three important parallels between children’s acquisition of negation terms, mental state terms, and temporal terms: (1) these terms do not refer to concrete objects; (2) children usually produce these words before they fully understand them; and (3) children’s understanding is affected by context and pragmatic factors. For example, negative sentences are only hard for children to process when they are pragmatically infelicitous (Nordmeyer and Frank, 2015; Reuter et al., 2018). de Villiers and de Villiers (2014) suggested that more conversation in rich social context allows the meanings for mental state words to emerge. Nelson (1996a,b) also emphasized the role of context in acquiring meanings for words referring to abstract entities. Children learn to use abstract words in contexts where others use them. Through using and interpreting words for abstract entities within their representation of familiar situations, children form a preliminary understanding of these words. As contexts and experiences accumulate, children’s understanding is refined and becomes connected to other representations in the construction of a conceptual network. At that time, their understanding is stable, decontexted, and conceptual. Nelson provided an insightful perspective on the constructive function of language, but also proposed that concepts are not wholly dependent upon linguistic expression. There must be some pre-linguistics representations onto which language can be mapped. The role of language in constructing abstract concepts in general, and the role of language in building temporal concepts in particular, needs to be addressed by more theoretical discussions and empirical investigations.

## How to Tease Apart and Measure Cognitive and Linguistic Components?

Although interrelations between language and conceptual development exist in many aspects of conceptual development, the connections are especially important and complicated for the concept of time. As a fundamental dimension of the universe, time is very abstract. Unlike number and space, it is difficult to instantiate with concrete entities. This makes language a crucial symbolic system for conceptual representation. At the same time, time itself is a conceptual tool to measure change and organize experience. Children’s temporal understanding develops in parallel with cognitive development and language development; it is also constructed through the interaction between cognitive processes and linguistic capacities. For a better understanding of the developmental trajectory of temporal concepts, it is necessary to tease apart and measure cognitive and linguistic components separately. However, practical challenges and difficulties exist in devising paradigms to assess children’s temporal cognition and temporal language separately (McCormack and Hoerl, 2008).

First, tasks that test children’s temporal language cannot easily avoid representational or reasoning demands. This issue is illustrated in research testing children’s understanding of *yesterday* and *tomorrow* (Tillman et al., 2015; Zhang and Hudson, 2018). In Zhang and Hudson (2018) *now* task, children needed to answer the question *What does it look like now*? based on sentences referring to an event occurring *yesterday* or *tomorrow*. To respond correctly, children had to first decode the temporality indicated by the sentence linguistically, and then parse the temporal relation between the referred event and the present. Children’s performances not only reflected their understanding of *yesterday* and *tomorrow*, but also demonstrated their temporal reasoning ability. Because forward temporal reasoning is easier than backward temporal reasoning, in their *now* task, answering the *now* question given an event occurring *yesterday* (*I carved the pumpkin yesterday. What does it look like now?*) was easier than answering the same question given an event occurring *tomorrow (I’m gonna carve the pumpkin tomorrow. What does it look like now?)*. Similar effects were evident in the study by Tillman et al. (2015). They showed 3- to 5-year-olds pictures of increasing events (e.g., a flower growing) and decreasing events (e.g., a snowman melting) and asked them to answer questions about *yesterday* and *tomorrow*. For example, for the event of a flower growing, they presented children a picture of flower today and asked them to select one picture from two alternatives to answer the questions, *What did the flower look like yesterday* or *What will the flower look like tomorrow?* Children performed better on questions requiring forward temporal reasoning (i.e., from today to tomorrow) than questions requiring backward temporal reasoning (i.e., from today to yesterday). Performances on these two tasks were affected by the reasoning processes required.

It would be very difficult to completely eliminate reasoning or memory in tasks aiming to measure language ability, but researchers can be aware of the effects of cognitive demands and try to minimize or test for their effects. For example, familiar settings and props can be used to reduce working memory and representation loads. Tasks can be designed to test for the effects of the cognitive demands required. For instance, in studying children’s understanding of *yesterday* and *tomorrow*, researchers can test and compare children’s comprehensions when the two terms are embedded in forward and backward reasoning settings, respectively.

Second, because temporal systems are abstract, we have to rely on language to express them, which means that it is difficult for researchers to only measure the cognitive components of temporal understanding. Many temporal reasoning and representation tasks rely heavily on children’s language comprehension. For example, Busby and Suddendorf (2010) investigated children’s ability to infer current physical and mental states based on past and future events. Children were told stories, each describing two characters. In the possession stories, one character had acquired an object in the past, and the other was going to acquire it in the future. In the knowledge stories, one character had already acquired the knowledge and the other was going to acquire it. Children were asked “which character has [the object]/knows [the knowledge] right now?” The stories were language heavy; each contained more than eight sentences, which required good language comprehension to understand, as wells as good memory skills to keep all of the relevant information in mind. More importantly, understanding of temporal expressions in the story (e.g., “Yesterday, Emma went shopping. When she went shopping she bought a new toothbrush” vs. “Tomorrow, Mindy is going shopping. When she is shopping she is going to buy a new toothbrush”) is the key for success in this task. If children simply do not know the meaning of temporal adverbs included (yesterday, tomorrow) and fail to parse or make use of information in past tense and future verb form, they would likely perform poorly. Therefore, their poor performance in this task could be due to the incorrect understanding of temporal expressions rather than to their inability to perform temporal reasoning. Results from this study showed that 4-year-olds’ performance was close to chance level. In a follow-up study, the authors simplified the stories by removing the temporal adverbs and adding auxiliaries *did* and *will* (e.g., “Emma went to the beach. She did take some shells home from the beach” vs. “Mindy is going to the beach. She will take some shells home from the beach”) and found that 4-year-olds’ performance significantly improved (above chance). This indicates that the way information is presented in language and children’s comprehension of linguistic information directly affect their temporal reasoning performance.

When studying temporal reasoning and representation, the use of language oftentimes cannot be avoided. In order to minimize language demands, future research focusing on temporal reasoning or judgments can make better use of pictures, props, and live or video demonstrations. For example, visible changes of objects over time (e.g., agents moving, plants growing) can be illustrated by using pictures or demonstrations together with linguistic descriptions. The contextual and visual accommodation may provide children alternatives to figure out the cognitive components asked by the tasks and reduce the demands for language as well as for memory. Researchers can also differentiate the events or scenarios used to study temporal concepts and relations in terms of familiarity. Familiar events can be used to detect the emergence of temporal reasoning and judgment abilities. Attention can be paid to whether children solve temporal reasoning or judgment problems based on their temporal cognitive skills or their memory of scripts for familiar events. In this case, memory factors can be measured and partialled out in data analyses. Unfamiliar or novel events can be used to test the proficiency of temporal cognitive skills. If children can apply the skills they use for familiar events to novel events, it shows that they have developed temporal cognitive skills that are generalizable and transferable.

Third, several cognitive processes of different complexity are often required when testing children’s temporal cognition due to varying tasks employed. Research on children’s temporal judgments has largely investigated three aspects: judgments about past–future status, judgments about distance of past/future events relative to the present, and placement of events along a timeline. The cognitive processes involved in each of these judgments are quite different. Past–future status is categorical judgment, which may only require a basic differentiation of the past and future. Temporal distance judgments are both categorical and continuous and require more cognitive processes, such as retrieving memory for the exact event time, representing conventional timeframes, and comparing the event time to the present in this mental timeframe. In general, past/future distance judgments are difficult for children; depending on tasks, they may also require cognitive flexibility or inhibitory control. For example, in Friedman (2003, 2005) studies, children were asked which of two cyclical events occurred longer before in the past, Christmas or the child’s birthday. The fact that both events happened in the past and will happen in the future makes the task ambiguous. Children might not fully understand what the task is asking for and simply respond based on the distances of events from present. Further, the question itself is not straightforward; in real life, when an annual event is upcoming, it is rare to be asked how long ago the previous one occurred. It is more cognitively adaptive to represent the upcoming occurrence as closer, rather than the previous one as farther away. To come up with the correct response, children had to closely attend to the question, inhibit the more salient representation, and switch to thinking about distances of past events. Given the complexity of the task, Friedman’s conclusion that children at age 4 or 5 still do not have a proper understanding of the distinction between the past and future calls for a careful re-examination.

To provide children with a visual representation of time, timeline-based tasks have employed a variety of forms of spatial representation. Researchers have used horizontal lines from left to right (e.g., Busby Grant and Suddendorf, 2009; Tillman et al., 2017), sagittal lines stretching away from the viewer (see Friedman, 2003 for a review), a line made of rectangles indicating time units (Hudson and Mayhew, 2011), and lines with markers indicating direction and scale (Busby Grant and Suddendorf, 2009; Tillman et al., 2017). These variations make it hard to compare children’s performances across studies and also raise interesting questions about the spatial representation of time. The limited research (Tillman et al., 2018) on mapping between time and space shows that children are initially flexible with spatial representations of time and most preschoolers do not represent time as a line spon-taneously. Their spatial representation of time becomes increasingly automatic and conventionalized in the early school years.

Similarly, research addressing temporal reasoning has used a variety of stimuli and methods. In research focused on sequencing (Nelson and Gruendel, 1981; Fivush and Mandler, 1985; Bauer and Shore, 1987; Bauer and Mandler, 1989), children are shown pictures of an event and are later asked to arrange randomly ordered pictures in the correct temporal order. The extent of children’s sequencing ability has been investigated by varying the types of events (e.g., familiar vs. unfamiliar; causal vs. arbitrary) and the manner of sequencing (e.g., forward vs. backward). Event representations, understanding of sequence, and memory are all required for reconstructing event sequences.

Another line of research has focused on children’s reasoning ability about temporal causal changes that requires cognitive abilities beyond event representation and sequencing. In this line of research, investigators were investigating children’s understanding of time as series of changes, specifically, their understanding of the causal pathway from the past to the present and the non-causal pathway from the future to the present. Friedman (unpublished, cited in Friedman, 2003) explicitly asked children the effect of a past or future event on the present (e.g., “Michelle had a birthday party yesterday. Can she know all the presents she got? Why or why not?”). Busby and Suddendorf (2010) told children stories about characters who did or will get/know something and asked them who had the thing or knew the information now. Tillman et al. (2015) showed children an event unfolding, such as a flower growing or a snowman melting, and asked them to identify what the item looked like in the past (yesterday) or would look like in the future (tomorrow) based their understanding of the event trajectory. The temporal-causal chain was especially important in McColgan and McCormack’s (2008) search and planning tasks. Children faced problems in contexts with many parameters and variables (e.g., the goal, the layout, the sequence, the direction, the time point). Temporal reasoning ability was necessary but not sufficient for them to solve the problems. They also needed to properly represent the goal and structure of the problem, be aware of contributing factors, temporally decenter themselves to envision the steps that needed to be taken forward or backward, and integrate steps and situations, either representational or imaginative, to make decisions. These studies differ in the complexity of the task context, and therefore call on different levels of other cognitive skills, such as working memory, cognitive flexibility, inhibition, and causal reasoning, to work together with temporal reasoning skills. This is perhaps one of the reasons that results vary even within temporal reasoning studies. Future research not only needs to disentangle cognitive and linguistic components in temporal understanding, but also needs to investigate elements of each component more systematically. For example, a series of tasks could be designed with increasing complexity, from processing basic temporal information to coordinating temporal and non-temporal factors in making inferences. Careful controls and contrasts could then be conducted across the series of tasks.

## Implications for Future Research

Psycholinguistic research has contributed much to our understanding of how children acquire temporal markers in language, but it has not fully explained the conceptual changes driven or brought on by language development. Researchers focusing on temporal representation and reasoning oftentimes utilize tasks that depend heavily on other cognitive abilities and knowledge (e.g., memory, cognitive flexibility, knowledge of annual holidays, etc.). The strengths and limitations of these two lines of research implicate several directions for future research.

In general, to better understand development, it would be helpful for researchers to first delineate a mature state of temporal concepts. The nature of time is perplexing; fundamental debates about the nature of time exist in physics (e.g., whether time exists independently of physical spacetime events or it is just a mere relationship of the causal ordering of events, Lobo, 2008) and philosophy (e.g., whether time is a series of events being either the past, present, or future or it is a series of events that one is “earlier than” another, McTaggart, 1908). Although conceptions of time may vary, psychologists interested in the cognitive understanding of time need to specify the key properties of temporal concepts under investigation. McCormack (2015) proposed three key properties of a mature concept of time. First, time is linear and unidirectional. It does not reoccur and cannot be revisited. Second, time is represented as unified, connected by before/after relations. Every time point is systematically related to every other point. Third, adults can think of time independent of events, that is, they can think about time points independent of events that have occurred or will occur. McCormack (2014) also hypothesized important developmental shifts in concepts of time from those grounded in script-like representations of repeated events, to concepts with distinct categories (happened vs. not yet), to a mature concept of event-independent time. This speculative account provides a way of thinking about development and calls for empirical investigation.

To capture emerging temporal concepts, studies focusing on language or cognitive processes need to adopt tasks that minimize cognitive demands for memory, inference, and inhibition. For example, instead of using verbally described vignettes in temporal language comprehension tasks, straightforward demonstrations of scenarios with child-friendly props could reduce cognitive loads and keep children engaged. Valian (2006) tested children’s understanding of temporal language by demonstrating and asking them about the familiar action of tying shoes, which effectively minimized memory and representation demands. Other linguistic factors, such as position of temporal words in a sentence and the telicity of verbs presented in task, should be unambiguous and well controlled. Another way to reduce task complexity would be to design tasks within well-known domains and based on events that are familiar to young children. For example, Friedman (1990) showed that children’s temporal reasoning was content-dependent; they were able to arrange familiar daily activities backwardly, but could not do the same for novel events, which demanded greater cognitive resources for memory, leaving fewer for inhibition (children had to inhibit their dominated response of reasoning in forward order). Once the initial starting point for temporal conceptualization is clear, researchers can explore the development of more advanced temporal reasoning by gradually increasing task complexity, for example, by including more temporal factors and inferential reasoning.

Third, multiple perspectives and various methods are needed to construct a full picture of conceptual development with respect to time. Previous research on psycholinguistics and cognitive processes has shed light on how children understand and reason about time, but more studies with well-controlled designs are needed to flesh out these two perspectives, and to facilitate conversations between the two. For example, future investigations could pay more attention to the contexts in which temporal terms emerge or new temporal terms/concepts are introduced to young children. Parents and children talk about events in their daily life and teachers and children talk about schedules and plans for activities in school settings. Adults can facilitate children’s language and conceptual learning in many ways. Research on parent-child talk about the past (Nelson and Fivush, 2004; Reese and Newcombe, 2007) showed that mothers’ elaborative reminiscing enhanced children’s autobiographical memory development. Research on parent–child talk about the future (Hudson, 2002, 2006) suggests that maternal time references contribute to children’s understanding and use of temporal terms. Future research could embrace more corpus analyses to find out the contextual factors that help children acquire temporal words and concepts of time. For example, in what context, do children start producing different types of temporal words? What social interactional cues and pragmatic cues are effective for early production? How does the quantity and quality of temporal language exposure affect children’s temporal language production and temporal understanding?

More research is also needed to compare and integrate findings from investigations of children’s production and comprehension of temporal language. This approach is exemplified in research on children’s production and comprehension of *no* and *not* in which children’s comprehension was measured by experimental tasks and children’s production was analyzed with respect to the Macarthur-Bates CDI production norms (Feiman et al., 2017). They found that children’s comprehension of the truth-functional *no* lagged behind their normal production of *no* by about a year, suggesting that the ability to map the concept of negation to the word *no* is developmentally challenging. Similarly, Sankaran (2011) investigated the influence of verb semantics on Tamil children’s acquisition of aspect markers using both a production task and a comprehension task. She found that children understood the imperfective marker before they actively used it, and although children frequently produced the perfective marker, their understanding of the function of the perfective marker was limited. This approach to comparing and integrating comprehension and production data can also be used to explicate the construction of temporal concepts. Ideally, future research should consider using within-subjects designs to study children’s comprehension and production of temporal language so that stronger claims can be made.

Efforts can also be made to design and employ on-line measures, such as preferential looking or eye tracking. Most previous research on temporal cognition and temporal language has adopted off-line measures, such as sentence-picture matching tasks, truth-value judgments, placement/sequencing task, act-out tasks, and question-after-story tasks. Online measures may be more sensitive and informative about the parsing/analyzing process. For example, Sekerina et al. (2004) tested children’s comprehension of pronouns using both on-line (eye tracking) and off-line (picture-selection) tasks. They found a dissociative pattern of performance across these two tasks. The eye-tracking task revealed a more adult-like competence than indicated by the picture selection task. Similarly, Brandt-Kobele and Höhle (2010) investigated 3- to 4-year-old German children’s comprehension of verb inflection as a cue to subject number using a preferential looking paradigm, where children did not have to perform a specific task, but instead their eye gaze was tracked to measure the comprehension of sentences with verb inflections. Using this paradigm, they found clear evidence that 3- to 4-year-olds were able to infer the number of subjects based on the inflectional information. When a similar task with both eye-tracking and pointing was conducted, Brandt-Kobele and Höhle (2010) found weaker evidence from children’s eye-movement data, and interestingly, no evidence from their pointing reactions. Children’s failure in selecting or pointing to the correct picture may be due to general task demands or to different stages of the interpretation process engaged by the on-line and the off-line measures (Trueswell and Gleitman, 2007). Although researchers are still debating whether preferential looking and picture selection tasks tap the same processes and what these cross-task discrepancies can tell us about comprehension-production asymmetry, for under-researched areas such as the development of temporal concepts, data from both on-line and off-line tasks could advance our understanding of the developmental trajectory.

Useful information and insights can also be obtained from the study of the development of related cognitive abilities and processes requiring temporal understanding. For example, an understanding of time is essential for autobiographical memory and future thinking. Remembering one’s own past implies an understanding of the past and a differentiation of past time points. Planning one’s own future implies an understanding of the future and a differentiation of future time points. Research has investigated the development of autobiographical memory, planning, and future thinking, but little attention has been paid to the extent that children understand the temporal concepts or temporal language presented in investigations. Considering children’s performances on autobiographical memory and future thinking tasks from the perspective of temporal understanding is helpful both for research in these areas themselves, but also for the study of temporal concepts, because an awareness of time is required and used for a pragmatic purpose in these tasks. Children may not fully understand the meaning of a temporal term or reason about temporal relations when asked explicitly, but it is possible that they can make use of their limited grasp of time when asked to recollect their past experiences and to imagine their future selves. Future research would benefit not only from disentangling the linguistic factors and cognitive processes in forming temporal concepts, but also from understanding how temporal concepts contribute to the development of other aspects of cognition and language.

## Author Contributions

MZ contributed to the conception and the writing of the manuscript. JH contributed to the writing of the manuscript by providing critical and valuable comments.

## Conflict of Interest Statement

The authors declare that the research was conducted in the absence of any commercial or financial relationships that could be construed as a potential conflict of interest.

## Footnotes

1 This review focuses on the emergence and development of temporal concepts from 2 to 6 years. However, children’s temporal understanding becomes more refined and sophisticated after age 6. Researchers have investigated how older children use temporal knowledge to improve their understanding of transformations in various domains (Montangero and Pownall, 1996), recall the time of past autobiographical events (Friedman, 2004) and construct life story narratives (Köber and Habermas, 2017), and develop an understanding of historical time (Thornton and Vukelich, 1988; Reisman and Wineburg, 2008).

2 Although evidence of young children’s understanding of before and after comes from this investigation of preschool children’s performance in a script-based task and from observations of preschool children’s spontaneous production of relational terms, such as *before, after, because, so, if, but, or* (French and Nelson, 1985), a flexible understanding of before and after, as tested by sentence comprehension tasks using more complex time clause structures (e.g., X before Y vs. before Y, X; Y after X vs. after X, Y), is not evident until age 12 (Pyykkönen and Järvikivi, 2012). More discussion of linguistic factors in the acquisition of the terms *before* and *after* can be found in Clark (1971) and Blything et al. (2015).

3 Temporal-causal connections are also observed in children’s personal narratives and stories, and children use temporal conjunctions (*then, next, first, before*, and *after*) to sequence actions within narratives (Hudson and Shapiro, 1991; Berman and Slobin, 2013). Because narrative production and story comprehension also depend on several other types of knowledge such as an understanding of episodic structure (a story schema), content knowledge, and metalinguistic knowledge, this literature is not included in our review.

## References

[B1] Ames L. B. (1946). The development of the sense of time in the young child. *J. Genet. Psychol.* 68 97–125. 10.1080/08856559.1946.10533358 21027252

[B2] Antinucci F. Miller R. (1976). How children talk about what happened. *J. Child Lang.* 3 167–189. 10.1017/S0305000900001434

[B3] Bartsch K. Wellman H. M. (1995). *Children Talk About the Mind.* New York, NY: Oxford University Press.

[B4] Bauer P. J. Mandler J. M. (1989). One thing follows another: effects of temporal structure on 1-to 2-year-olds’ recall of events. *Dev. Psychol.* 25 197–206. 10.1037/0012-1649.25.2.197

[B5] Bauer P. J. Mandler J. M. (1992). Putting the horse before the cart: the use of temporal order in recall of events by one-year-old children. *Dev. Psychol.* 28 441–452. 10.1037/0012-1649.28.3.441

[B6] Bauer P. J. Shore C. M. (1987). Making a memorable event: effects of familiarity and organization on young children’s recall of action sequences. *Cogn. Dev.* 2 327–338. 10.1016/S0885-2014(87)80011-4

[B7] Berman R. A. Slobin D. I. (2013). *Relating Events in Narrative: A Crosslinguistic Developmental Study.* London: Psychology Press 10.4324/9780203773512

[B8] Bloom L. (1970). *Language Development: Form and Function in Emerging Grammars.* Cambridge, MA: MIT Press.

[B9] Blything L. Davies R. Cain K. (2015). Young children’s comprehension of temporal relations in complex sentences: the influence of memory on performance. *Child Dev.* 86 1922–1934. 10.1111/cdev.12412 26309072 PMC4975597

[B10] Brandt-Kobele O. C. Höhle B. (2010). What asymmetries within comprehension reveal about asymmetries between comprehension and production: the case of verb inflection in language acquisition. *Lingua* 120 1910–1925. 10.1016/j.lingua.2010.02.008

[B11] Bronckart J. P. Sinclair H. (1973). Time, tense and aspect. *Cognition* 2 107–130. 10.1016/0010-0277(72)90032-7

[B12] Busby J. Suddendorf T. (2005). Recalling yesterday and predicting tomorrow. *Cogn. Dev.* 20 362–372. 10.1016/j.cogdev.2005.05.002

[B13] Busby J. G. Suddendorf T. (2010). Young children’s ability to distinguish past and future changes in physical and mental states. *Br. J. Dev. Psychol.* 28 853–870. 10.1348/026151009X482930 21121471

[B14] Busby Grant J. Suddendorf T. (2009). Preschoolers begin to differentiate the times of events from throughout the lifespan. *Eur. J. Dev. Psychol.* 6 746–762. 10.1080/17405620802102947

[B15] Busby Grant J. Suddendorf T. (2011). Production of temporal terms by 3-, 4-, and 5-year-old children. *Early Child. Res. Q.* 26 87–95. 10.1016/j.ecresq.2010.05.002

[B16] Carni E. French L. A. (1984). The acquisition of before and after reconsidered: what develops? *J. Exp. Child Psychol.* 37 394–403. 10.1016/0022-0965(84)90011-0

[B17] Christie S. Gentner D. (2014). Language helps children succeed on a classic analogy task. *Cogn. Sci.* 38 383–397. 10.1111/cogs.12099 24215433

[B18] Clark E. (1971). On the acquisition of the meaning of before and after. *J. Verbal Learn. Verbal Behav.* 10 266–275. 10.1016/S0022-5371(71)80054-3

[B19] Clark E. V. (1995). *The Lexicon in Acquisition.* Cambridge: Cambridge University Press.

[B20] de Villiers J. G. de Villiers P. A. (2003). “Language for thought: coming to understand false beliefs,” in *Language in Mind: Advances in the Study of Language and Thought* eds Gentner D. Goldin-Meadow S. (Cambridge, MA: MIT Press) 335–384.

[B21] de Villiers J. G. de Villiers P. A. (2014). The role of language in Theory of Mind development. *Top. Lang. Disord.* 34 313–328. 10.1097/TLD.0000000000000037

[B22] Di Paolo M. Smith C. (1978). “Cognitive and linguistic factors in language acquisition: the use of temporal and aspectual expressions,” in *The Development of Meaning. Pedolinguistic Series II* ed. French P. (Tokyo: Bunka Hy-anon).

[B23] Feiman R. Mody S. Sanborn S. Carey S. (2017). What do you mean, no? Toddlers’ comprehension of logical “no” and “not”. *Lang. Learn. Dev.* 13 430–450. 10.1080/15475441.2017.1317253

[B24] Fivush R. Mandler J. M. (1985). Developmental changes in the understanding of temporal sequence. *Child Dev.* 56 1437–1446. 10.2307/1130463 4075867

[B25] French L. A. Nelson K. (1985). *Young Children’s Knowledge of Relational Terms: Some Ifs, Ors and Buts.* New York, NY: Springer-Verlag 10.1007/978-1-4613-8581-3

[B26] Friedman W. J. (1990). Children’s representations of the pattern of daily activities. *Child Dev.* 61 1399–1412. 10.2307/1130751 2245733

[B27] Friedman W. J. (2000). The development of children’s knowledge of the times of future events. *Child Dev.* 71 913–932. 10.1111/1467-8624.00199 11016556

[B28] Friedman W. J. (2002). Children’s knowledge of the future distances of daily activities and annual events. *J. Cogn. Dev.* 3 333–356. 10.1207/S15327647JCD0303_4

[B29] Friedman W. J. (2003). The development of a differentiated sense of the past and the future. *Adv. Child Dev. Behav.* 31 229–269. 10.1016/S0065-2407(03)31006-7 14528663

[B30] Friedman W. J. (2004). Time in autobiographical memory. *Soc. Cogn.* 22 591–605. 10.1521/soco.22.5.591.50766

[B31] Friedman W. J. (2005). Developmental and cognitive perspectives on humans’ sense of the times of past and future events. *Learn. Motiv.* 36 145–158. 10.1016/j.lmot.2005.02.005

[B32] Friedman W. J. Gardner A. C. Zubin N. R. (1995). Children’s comparisons of the recency of two events from the past year. *Child Dev.* 66 970–983. 10.2307/1131792

[B33] Friedman W. J. Kemp S. (1998). The effects of elapsed time and retrieval on young children’s judgments of the temporal distances of past events. *Cogn. Dev.* 13 335–367. 10.1016/S0885-2014(98)90015-6

[B34] Gelman R. Bullock M. Meck E. (1980). Preschoolers’ understanding of simple object transformations. *Child Dev.* 51 691–699. 10.2307/1129454 7418506

[B35] Gelman S. A. Coley J. D. (1991). “Language and categorization: the acquisition of natural kind terms,” in *Perspectives on Language and Thought: Interrelations in Development* eds Gelman S. A. Byrnes J. P. (Cambridge: Cambridge University Press) 146–196. 10.1017/CBO9780511983689.006

[B36] Gentner D. (2003). “Why we’re so smart?,” in *Language in Mind: Advances in the Study of Language and Thought* eds Gentner D. Goldin-Meadow S. (Cambridge, MA: MIT Press) 195–235.

[B37] Grimm A. Müller A. Hamann C. Ruigendijk E. (eds) (2011). *Production-Comprehension Asymmetries in Child Language* Vol. 43. Berlin: Walter de Gruyter 10.1515/9783110259179

[B38] Harner L. (1975). Yesterday and tomorrow: development of early understanding of the terms. *Dev. Psychol.* 11 864–865 10.1037/0012-1649.11.6.864

[B39] Harner L. (1981). Children talk about the time and aspect of actions. *Child Dev.* 52 498–506. 10.1017/S0305000913000135 23714767

[B40] Harner L. (1982). “Talking about the past and the future,” in *The Developmental Psychology of Time* ed. Friedman W. J. (New York, NY: Academic Press) 141–169.

[B41] Hendriks P. (2014). *Asymmetries Between Language Production and Comprehension.* Dordrecht: Springer 10.1007/978-94-007-6901-4

[B42] Hendriks P. Koster C. (2010). Production/comprehension asymmetries in language acquisition. *Lingua* 120 1887–1897. 10.1016/j.lingua.2010.02.002

[B43] Herriot P. (1969). The comprehension of tense by young children. *Child Dev.* 40 103–110. 10.2307/1127159

[B44] Hudson J. A. Shapiro L. R. (1991). “From knowing to telling: the development of children’s scripts, stories, and personal narratives,” in *Developing Narrative Structure* eds McCabe A. Peterson C. (London: Psychology Press) 89–136.

[B45] Hudson J. A. (2002). “Do you know what we’re going to do this summer?”: mothers’ talk to preschool children about future events. *J. Cogn. Dev.* 3 49–71. 10.1207/S15327647JCD0301_4

[B46] Hudson J. A. (2006). The development of future time concepts through mother-child conversation. *Merrill Palmer Q.* 52 70–95. 10.1353/mpq.2006.0005

[B47] Hudson J. A. Mayhew E. M. Y. (2011). Children’s temporal judgments for autobiographical past and future events. *Cogn. Dev.* 26 331–342. 10.1016/j.cogdev.2011.09.005

[B48] Klein W. (2009). “How time is encoded,” in *The Expression of Time* eds Klein W. Li P. (Berlin: De Gruyter Mouton) 1–43.

[B49] Köber C. Habermas T. (2017). Development of temporal macrostructure in life narratives across the lifespan. *Discourse Process.* 54 143–162. 10.1080/0163853X.2015.1105619

[B50] Kuczaj S. A. II (1977). The acquisition of regular and irregular past tense forms. *J. Verbal Learn. Verbal Behav.* 16 589–600. 10.1016/S0022-5371(77)80021-2

[B51] Lobo F. S. (2008). “Nature of time and causality in physics,” in *Psychology of time* ed. Grondin S. (Bingley: Emerald Group Publishing Limited) 395–422.

[B52] Lohse K. Kalitschke T. Ruthmann K. Rakoczy H. (2015). The development of reasoning about the temporal and causal relations among past, present, and future events. *J. Exp. Child Psychol.* 138 54–70. 10.1016/j.jecp.2015.04.008 26037402

[B53] Mandler J. (1986). “The development of event memory,” in *Human Memory and Cognitive Capabilities: Mechanisms and Performance* eds Klix F. Hagendoft H. (Toronto, CA: Elsevier Science Ltd) 459–467.

[B54] Markman E. M. (1991). “The whole-object, taxonomic, and mutual exclusivity assumptions as initial constraints on word meanings,” in *Perspectives on Language and Thought: Interrelations in Development* eds Gelman S. A. Byrnes J. P. (Cambridge Cambridge University Press) 72–106.

[B55] McColgan K. L. McCormack T. (2008). Searching and planning: young children’s reasoning about past and future event sequences. *Child Dev.* 79 1477–1497. 10.1111/j.1467-8624.2008.01200.x 18826537

[B56] McCormack T. Hoerl C. (2001). “The child in time: temporal concepts and self-consciousness in the development of episodic memory,” in *The Self in Time: Developmental Perspectives* eds Moore C. Lemmon K. Skene K. (London: Psychology Press) 203–227.

[B57] McCormack T. (2014). Three types of temporal perspective: characterizing developmental changes in temporal thought. *Ann. N. Y. Acad. Sci.* 1326 82–89. 10.1111/nyas.12504 25312781

[B58] McCormack T. (2015). “The development of temporal cognition,” *Handbook of Child Psychology and Developmental Science*: Cognitive Processes Vol. 2 eds Lerner R. M. Liben L. S. Mueller U. (Hoboken, NY: Wiley-Blackwell) 624–670. 10.1002/9781118963418.childpsy215

[B59] McCormack T. Hoerl C. (1999). Memory and temporal perspective: the role of temporal frameworks in memory development. *Dev. Rev.* 19 154–182. 10.1006/drev.1998.0476

[B60] McCormack T. Hoerl C. (2008). Temporal decentering and the development of temporal concepts. *Lang. Learn.* 58 89–113. 10.1111/j.1467-9922.2008.00464.x 24411117

[B61] McTaggart J. E. (1908). The unreality of time. *Mind* 17 457–474. 10.1093/mind/XVII.4.457

[B62] Montangero J. Pownall T. T. (1996). *Understanding Changes in Time: The Development of Diachronic Thinking in 7-to 12-Year-Old Children.* Milton Park: Taylor & Francis.

[B63] Nelson K. (1977). *Cognitive Development and the Acquisition of Concepts. Schooling and the Acquisition of Knowledge.* Hillsdale, NJ: Erlbaum 215–238.

[B64] Nelson K. (1989). “Monologue as the linguistic construction of self in time,” in *Narratives from the crib* ed. Nelson K. (Cambridge, MA: Harvard University Press) 284–308.

[B65] Nelson K. (1991). “The matter of time: Interdependencies between language and thought in development,” in *Perspectives on Language and Thought: Interrelations in Development* eds Gelman S. A. Byrnes J. P. (Cambridge: Cambridge University Press) 278–318.

[B66] Nelson K. (1996a). “The emergence of the projective mind,” in *Language in Cognitive Development: The Emergence of the Mediated Mind* ed. Nelson K. (New York, NY: Cambridge University Press) 292–322. 10.1017/CBO9781139174619.011

[B67] Nelson K. (1996b). “The emergence of the temporal mind,” in *Language in Cognitive Development: The Emergence of the Mediated Mind* ed. Nelson K. (New York, NY: Cambridge University Press) 259–291. 10.1017/CBO9781139174619.010

[B68] Nelson K. Fivush R. (2004). The emergence of autobiographical memory: a social cultural developmental theory. *Psychol. Rev.* 111 486–511. 10.1037/0033-295X.111.2.486 15065919

[B69] Nelson K. Gruendel J. (1981). Generalized event representations: basic building blocks of cognitive development. *Adv. Dev. Psychol.* 1 130–158.

[B70] Nordmeyer A. E. Frank M. C. (2015). “Negation is only hard to process when it is pragmatically infelicitous,” in *Proceedings of the 37th Annual Meeting of the Cognitive Science Society* Austin, TX 23–25.

[B71] O’Connell B. G. Gerard A. B. (1985). Scripts and scraps: the development of sequential understanding. *Child Dev.* 56 671–681. 10.2307/1129757 2258692

[B72] Papafragou A. Musolino J. (2003). Scalar implicatures: experiments at the semantics–pragmatics interface. *Cognition* 86 253–282. 10.1016/S0010-0277(02)00179-8 12485740

[B73] Pawlak A. Oehlrich J. S. Weist R. M. (2006). Reference time in child English and Polish. *First Lang.* 26 281–297. 10.1177/0142723706059447

[B74] Piaget J. (1968). *Six Psychological Studies.* New York, NY: Vintage Books.

[B75] Povinelli D. J. Landry A. M. Theall L. A. Clark B. R. Castille C. M. (1999). Development of young children’s understanding that the recent past is causally bound to the present. *Dev. Psychol.* 35 1426–1439. 10.1037/0012-1649.35.6.1426 10563732

[B76] Pyykkönen P. Järvikivi J. (2012). Children and situation models of multiple events. *Dev. Psychol.* 48 521–529. 10.1037/a0025526 21967560

[B77] Reese E. Newcombe R. (2007). Training mothers in elaborative reminiscing enhances children’s autobiographical memory and narrative. *Child Dev.* 78 1153–1170. 10.1111/j.1467-8624.2007.01058.x 17650131

[B78] Reichenbach H. (1947). *Elements of Symbolic Logic.* New York, NY: Dover Publications, Inc.

[B79] Reinhart T. (2004). The processing cost of reference set computation: acquisition of stress shift and focus. *Lang. Acquis.* 12 109–155. 10.1207/s15327817la1202_1

[B80] Reisman A. Wineburg S. (2008). Teaching the skill of contextualizing in history. *Soc. Stud.* 99 202–207. 10.3200/TSSS.99.5.202-207

[B81] Reuter T. Feiman R. Snedeker J. (2018). Getting to no: pragmatic and semantic factors in two-and three-year-olds’ understanding of negation. *Child Dev.* 89 e364–e381. 10.1111/cdev.12858 28617962

[B82] Sachs J. (1979). Topic selection in parent-child discourse. *Discourse Process.* 2 145–153. 10.1080/01638537909544460 27136194

[B83] Sachs J. (1983). Talking about the there and then: the emergence of displaced reference in parent-child discourse. *Child. Lang.* 4 1–28.

[B84] Sankaran L. (2011). “Testing the aspect hypothesis in child Tamil,” in *Production-Comprehension Asymmetries in Child Language* eds Grimm A. Müller A. Hamann C. Ruigendijk E. (Berlin: Walter de Gruyter) 17–38.

[B85] Sekerina I. A. Stromswold K. Hestvik A. (2004). How do adults and children process referentially ambiguous pronouns? *J. Child Lang.* 31 123–152. 10.1017/S0305000903005890 15053087

[B86] Thornton S. J. Vukelich R. (1988). Effects of children’s understanding of time concepts on historical understanding. *Theory Res. Soc. Educ.* 16 69–82. 10.1080/00933104.1988.10505556

[B87] Tillman K. Cheung P. Tulagan N. Barner D. (2015). “Do some languages tell time better than others? Acquisition of time words in English- and Chinese-speaking children,” in *Poster Presented at the Biennial Meeting of the Cognitive Development Society* Columbus, OH.

[B88] Tillman K. A. Barner D. (2015). Learning the language of time: children’s acquisition of duration words. *Cogn. Psychol.* 78 57–77. 10.1016/j.cogpsych.2015.03.001 25867093

[B89] Tillman K. A. Marghetis T. Barner D. Srinivasan M. (2017). Today is tomorrow’s yesterday: children’s acquisition of deictic time words. *Cogn. Psychol.* 92 87–100. 10.1016/j.cogpsych.2016.10.003 27914312

[B90] Tillman K. A. Tulagan N. Fukuda E. Barner D. (2018). The mental timeline is gradually constructed in childhood. *Dev. Sci.* 21:e12679. 10.1111/desc.12679 29749676

[B91] Trueswell J. C. Gleitman L. R. (2007). “Learning to parse and its implications for language acquisition,” in *Oxford Handbook of Psycholinguistics* ed. Gaskell G. (Oxford: Oxford Univeristy Press) 635–656.

[B92] Valian V. (1991). Syntactic subjects in the early speech of American and Italian children. *Cognition* 40 21–81. 10.1016/0010-0277(91)90046-7 1786672

[B93] Valian V. (2006). Young children’s understanding of present and past tense. *Lang. Learn. Dev.* 2 251–276. 10.1007/s11764-015-0465-8 26123806

[B94] Valian V. Aubry S. (2005). When opportunity knocks twice: two-year-olds’ repetition of sentence subjects. *J. Child Lang.* 32 617–641. 10.1017/S0305000905006987 16220637

[B95] van Hout A. (2007). “Optimal and Non-Optimal Interpretations in the Acquisition of Dutch Past Tenses”, in *Proceedings of the 2nd Conference on Generative Approaches to Language Acquisition North America (GALANA)* eds. Belikova A. Meroni L. Umeda M. (Somerville, MA: CascadillaProceedings Project) 159–170.

[B96] Vygotsky L. (1962). *Thought and Language.* Cambridge, MA: MIT press 10.1037/11193-000

[B97] Wagner L. (2001). Aspectual influences on early tense comprehension. *J. Child Lang.* 28 661–681 11797543 10.1017/s0305000901004792

[B98] Waxman S. R. (1991). “Convergences between semantic and conceptual organization in the preschool years,” in *Perspectives on Language and Thought: Interrelations in Development* eds Gelman S. A. Byrnes J. P. (Cambridge: Cambridge University Press) 107–145.

[B99] Waxman S. R. (2004). “Everything had a name, and each name gave birth to a new thought: links between early word learning and conceptual organization,” in *Weaving a Lexicon* eds Hall D. G. Waxman S. R. (Cambridge, MA: MIT Press) 295–335.

[B100] Waxman S. R. Markow D. B. (1995). Words as invitations to form categories: evidence from 12- to 13-month-old infants. *Cogn. Psychol.* 29 257–302. 10.1006/cogp.1995.1016 8556847

[B101] Weist R. Wysocka H. Lyytinen P. (1991). A cross-linguistic perspective on the development of temporal systems. *J. Child Lang.* 18 67–92. 10.1017/S0305000900013301 2010506

[B102] Weist R. M. (1989). “Time concepts in language and thought: filling the Piagetian void from two to five years,” in *Time and Human Cognition: A Life-Span Perspective* eds Levin I. Zakay D. (Oxford: North Holland) 63–118.

[B103] Zhang M. Hudson J. A. (2018). Children’s understanding of yesterday and tomorrow. *J. Exp. Child Psychol.* 170 107–133. 10.1016/j.jecp.2018.01.010 29462727

